# Effective Blocking of Microbial Transcriptional Initiation by dCas9-NG-Mediated CRISPR Interference

**DOI:** 10.4014/jmb.2008.08058

**Published:** 2020-09-22

**Authors:** Bumjoon Kim, Hyun Ju Kim,, Sang Jun Lee

**Affiliations:** Department of Systems Biotechnology, Chung-Ang University, Anseong 17546, Republic of Korea

**Keywords:** CRISPR interference, PAM sequence, dCas9-NG, *gal* promoter, D-galactose

## Abstract

CRISPR interference (CRISPRi) has been developed as a transcriptional control tool by inactivating the DNA cleavage ability of Cas9 nucleases to produce dCas9 (deactivated Cas9), and leaving dCas9 the ability to specifically bind to the target DNA sequence. CRISPR/Cas9 technology has limitations in designing target-specific single-guide RNA (sgRNA) due to the dependence of protospacer adjacent motif (PAM) (5'-NGG) for binding target DNAs. Reportedly, Cas9-NG recognizing 5'-NG as the PAM sequence has been constructed by removing the dependence on the last base G of PAM through protein engineering of Cas9. In this study, a dCas9-NG protein was engineered by introducing two active site mutations in Cas9-NG, and its ability to regulate transcription was evaluated in the *gal* promoter in *E. coli*. Analysis of cell growth rate, D-galactose consumption rate, and *gal* transcripts confirmed that dCas9-NG can completely repress the promoter by recognizing DNA targets with PAM of 5'-NGG, NGA, NGC, NGT, and NAG. Our study showed possible PAM sequences for dCas9-NG and provided information on target-specific sgRNA design for regulation of both gene expression and cellular metabolism.

## Introduction

CRISPR (clustered regularly interspaced short palindromic repeats) is an adaptive immune system of bacteria and archaea [1]. The microorganism that survived from the invasion of bacteriophages can memorize the DNA fragments of the phage in the CRISPR loci in the microbial genome [2]. When the phage re-infiltrated, the single-guide RNA (sgRNA) transcribed from the stored CRISPR loci guides the Cas9 nuclease to the nucleic acids of the phage, and Cas9-sgRNA complex can specifically recognize and cleave the phage DNA or RNA [3, 4]. For the Cas9-sgRNA complex to recognize and cleave DNA, there must be a PAM (protospacer adjacent motif) sequence near the target sequence [5]. PAM sequence exists to cut only the phage DNA as a target, and not the memorized DNA fragments derived from the phage, ultimately preventing bacterial chromosome from recognizing as a target sequence [6, 7].

From a bacterial adaptive immune system, CRISPR/Cas technology has been evolved and used for accurate and efficient genome editing [8-11]. However, there is still a drawback that the PAM sequence should recognized along with the designed DNA target sequence. Two different directions have been studied to solve the limitation of the PAM of Cas9, a specific DNA sequence (5'-NGG). First, other CRISPR/Cas systems with different PAM sequences have been discovered. For example, the CRISPR/Cas12a system, also known as Cfp1, was found in *Francisella novicida*, and has 5'-TTTN as the PAM sequence [12], and CasX (Cas12e) has 5’-TTCN as the PAM sequence [13]. Second, PAM dependence has been relieved by protein engineering of Cas9 nuclease. Reportedly, site specific mutagenesis of three amino acids allowed Cas9 nuclease to have 5’-NGA as PAM sequence [14, 15]. Seven amino acid changes (R1335V, L1111R, D1135V, G1218R, E1219F, A1322R, and T1337R) created Cas9-NG nuclease to recognize broad PAM sequence (5’-NG) so that genome editing was successful in mammalian cells [16].

Besides, CRISPR interference (CRISPRi) has been developed for regulation of gene expression by removing nuclease activity in Cas9 protein. dCas9 (deactivated Cas9) was generated by introducing point mutations (D10A, H840A) into the HNH and RuvC domains in Cas9, which are responsible for nuclease activity. Since dCas9-sgRNA complex can specifically bind to target DNAs such as promoters, and repress the transcription, CRISPRi can be used for metabolic engineering by tuning metabolic pathways [17-19]. For example, the reduction of pgi and *pck* expression by CRISPRi was applied to the production of L-lysine in *Corynebacterium glutamicum* [20]. Overproduction of succinate was achieved in cyanobacterial cells by regulating genetic network through CRISPRi [21]. In addition, CRISPRi system was used to study toxicity, and drug resistance in pathogenic *Staphylococcus aureus* [22].

Although CRISPRi has been successfully harnessed in the metabolic engineering of diverse microorganisms, the PAM dependence still exists in designing the target DNA sequences. Unlike Cas9 protein that recognizes and cleaves the target position, the dCas9 protein can regulate gene expression only by attaching to the DNA target. It was thought that Cas9 and dCas9 may have different PAM dependencies. Previously, we confirmed that 5'-NGA, NAG, and NTG can work as a PAM sequence in dCas9 in addition to 5'-NGG [23].

In this study, we constructed dCas9-NG from Cas9-NG by removing nuclease activity, and compared both the regulation of the *gal* promoter and galactose metabolism using CRISPR/dCas9-NG with various PAM sequences. Information on the tolerance and limits of PAM in dCas9-NG will aid in elaborate gene expression in cells and metabolic biotechnology.

## Materials and Methods

### Strains and Culture Conditions

*Escherichia coli* DH5α, and MG1655 were used as a cloning host and a dCas9-NG expression strain, respectively (Table 1). *E. coli* DH5α cells were grown in LB (LPS solution, Cat. No. LB-05, Korea) medium, and MG1655 derivatives were grown in LB broth as starter cultures, and transferred to M9 broth containing sodium succinate (Sigma-Aldrich, Cat. No. 14160, USA) for main culture (see below for details). MacConkey agar (BD Dico, Cat. No. 281810, USA) plates containing D-galactose (0.5%) (Samchun chemicals, Cat. No. G0476, Korea) were used to check whether cells utilize D-galactose as carbon sources. If needed, antibiotics such as ampicillin (50 µg/ml), kanamycin (25 µg/ml), and spectinomycin (75 µg/ml) were added. L-arabinose (final 1 mM) (TCI, Cat. No. A0515, Japan) was added for the expression of dCas9-NG proteins in cells.

### Genomic Integration of *dcas9-NG*

To generate the *dcas9-NG*-KmR cassette for genomic integration, we fused the following three DNA fragments using overlap PCR: First, 5’-part (3 kb) of *dcas9-NG* gene was amplified using the genomic DNA of HK1060 strain as a template and primer pairs (P1 and P2). Second, 3’-part (837 bp) of *cas9-NG* gene was codon-optimized using Codon Optimization Tool (https://sg.idtdna.com/pages/tools/codon-optimization-tool) and chemically synthesized (Integrated DNA Technologies, USA). Third, kanamycin resistance marker was obtained by PCR of KmR-FRT fragment from KEIO collection using primer pairs (P3 and P4). The *dcas9-NG*-KmR cassette was purified and electroporated into L-arabinose induced *E. coli* MG1655 harboring pKD46 to generate *E. coli* HK1160 (Δ*araBAD:P_BAD_*-*dcas9-NG*-KmR). Then, plasmid pKD46 was cured at 42°C, and the integration of the *dcas9-NG* gene, located downstream of the *P_BAD_* promoter, into the genome of *E. coli* HK1160 was confirmed by Sanger sequencing.

### Construction of sgRNA Plasmids

sgRNA expression plasmids were designed to recognize the *gal* promoter as target sequences, as described previously [23]. Plasmid pHK459 was used as a template to amplify 0.9 and 1.2 kb fragments with P5 and P6 plus P7 and P8 primer pairs. The two fragments were isothermally assembled to make pBJ035 using Gibson Assembly Master Mix (NEB, Cat. No. E2611, USA). Other sgRNA plasmids (pBJ036 and pBJ037) were produced in the same manner. All the sgRNA plasmids listed in Table 1 were confirmed by Sanger sequencing and transformed into HK1060 and HK1160 cells for further experiments.

### Colony Color Assay

Each sgRNA plasmids were transformed into HK1060 and HK1160 cells, and transformant cells were streaked on MacConkey agar containing spectinomycin (final 75 µg/ml) and D-galactose (0.5%) as a carbon source at 37°C for 12 h. When L-arabinose (final 1 mM) was added on the MacConkey agar containing D-galactose, the transformant cells can express *dcas9* (or *dcas9-NG*) gene to make dCas9-sgRNA (or dCas9-NG-sgRNA) complex. If protein-sgRNA complex can repress the *gal* promoter, and cells are not able to use D-galactose, the color of colonies remains white. Conversely, if cells can utilize D-galactose due to a failure to repress the *gal* promoter, the color turns red.

### Growth Assay and Metabolite Analysis

HK1160 cells with sgRNA plasmids were grown in LB broth containing spectinomycin at 37°C for 15 h. 1.0% of cells were inoculated in 25 ml of M9 minimal medium containing sodium succinate (final 20 mM) with or without L-arabinose (final 1mM), and spectinomycin in 250 ml flasks. Six hours after the beginning of the flask culture, D-galactose (final 20 mM) was added. Cell growth was monitored by measuring optical density at 600 nm of culture broths every 3 h using an Ultrospec 8000 spectrophotometer (GE Healthcare, Sweden). The concentration of metabolites such as D-galactose and succinate in the culture was determined by high-performance liquid chromatography (RID-10A RI monitor, Shimadzu, Japan) with an Aminex HPX-87H column (300×7.8 mm, BioRad) as described previously [23, 24]. After centrifugation of the cell culture broth, the supernatant was filtered by a 0.2 µm syringe filter. The column was isocratically eluted at 47°C with a flow rate of 0.5 ml/min using 0.01 N H_2_SO_4_.

### The *gal* Transcript Analysis

Transcription levels of *galE* gene were monitored using quantitative real-time PCR (RT-qPCR) to confirm whether dCas9-NG-sgRNA complex can repress the *gal* promoter. Three hours after D-galactose addition, total RNAs were isolated using the RNeasy Mini kit (Cat. No. 74104; Qiagen, Germany). Primers for the *galE* transcript was designed at the Universal Probe Library Assay Design Center (http://lifescience.roche.com) and listed in Table 2. Five nanograms of each total RNAs were mixed with reagents in the RealHelix qPCR kit (Nanohelix, Cat. No. QP2-S500, Korea), and RT-qPCR reactions were performed un the following conditions: : cDNA synthesis (50°C, 40 min); denaturation (95°C, 12 min); amplification for 40 cycles (95°C, 20 sec; 60°C, 1 min); measuring melt curve (65°C to 95 °C: Increment 0.5°C, 5 sec). Fluorescence signals in the RT-qPCR were analyzed using a CFX96 Touch (Bio-Rad, USA), and the raw fluorescence data were normalized against the expression level of 16S ribosomal RNA. The relative abundance of *galE* (374–453 base region from +1 start codon) in the presence of L-arabinose were divided by mRNA levels of the corresponding genes in the absence of L-arabinose.

## Results

### The Repression of *gal* Operon by dCas9-NG and sgRNA Complex

**Fig. 1:**
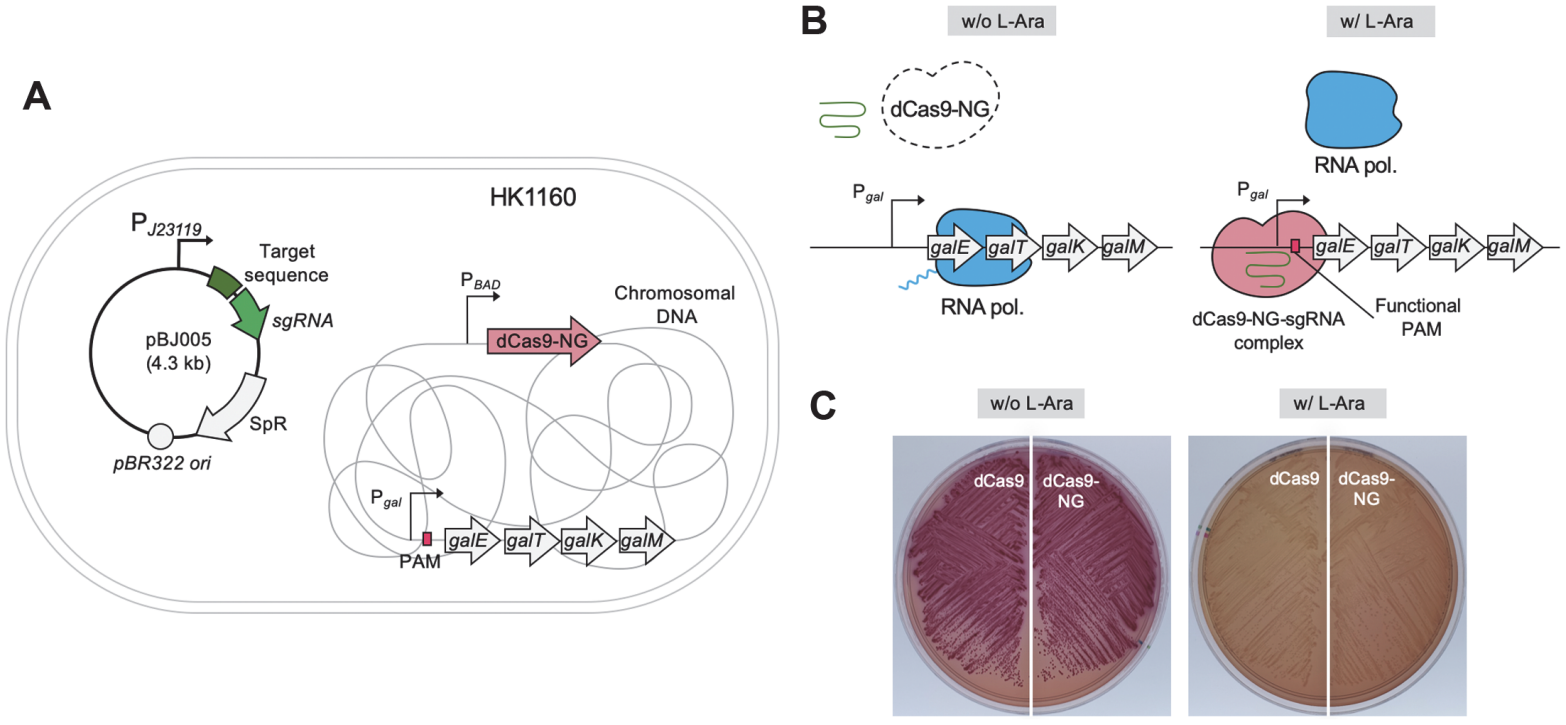
Construction of CRISPRi system with dCas9-NG in *E. coli*. (**A**) Chromosomal construction of *Escherichia coli* HK1160 (*P_BAD_*-dCas9-NG-KmR) and structure of sgRNA plasmid. dCas9-NG protein is expressed by L-arabinose-inducible *P_BAD_* promoter. sgRNA is constitutively expressed by *P_j23119_* promoter. (**B**) Regulation of *gal* operon transcription by dCas9-NG and sgRNA complex. In the absence of L-arabinose, dCas9-NG is not expressed and *gal* transcription can be initiated by RNA polymerase. In the presence of L-arabinose, dCas9-NG is expressed and dCas9-NG-sgRNA complex recognizes target DNA adjacent to functional PAM sequence. (**C**) Switch on/off of L-arabinose-inducible CRISPRi. In the presence of L-arabinose, Cas9 and dCas9 are expressed to repress the expression of D-galactose metabolizing enzymes (white colonies on MacConkey agar containing D-galactose). In the absence of L-arabinose, cells can utilize D-galactose (red colonies).

The *dcas9-NG* gene was produced by the fusion of 5’-fragment (3.0 kb) from *dcas9* gene and 3’- synthesized fragment (0.8 kb). We optimized the codon of the 3’-DNA fragment, because the original *cas9-NG* gene carrying seven point mutations (encoding R1335V, L1111R, D1135V, G1218R, E1219F, A1322R, and T1337R) were designed for human cells [16]. The newly synthesized *dcas9-NG* gene was inserted downstream of the L-arabinose inducible *P_BAD_* promoter in the genome of *E. coli* MG1655, which was named *E. coli* HK1160 strain (Fig. 1A).

Next, sgRNA plasmid such as pBJ005 was transformed into HK1160 cells. The *gal* promoter was designed as the DNA target of dCas9-NG-sgRNA complex in HK1160 cells. In the absence of L-arabinose, the expression of *dcas9-NG* gene is not induced and the transcription the *gal* operon is expressed to make D-galactose metabolizing enzymes (Fig. 1B). In the presence of L-arabinose, it is assumed that dCas9-NG-sgRNAs complex can bind to the DNA target with functional PAM, and repress the *gal* promoter. If the PAM sequence is not functional, dCas9-NG-sgRNAs complex cannot bind to the DNA target in the *gal* promoter, resulting the failure of repression of the *gal* operon.

D-galactose metabolism of HK1060 (dCas9) carrying pBJ005 and HK1160 (dCas9-NG) carrying the same pBJ005 plasmid was compared with and without L-arabinose on MacConkey D-galactose agar plate (Fig. 1C). The pBJ005 plasmid was designed to express sgRNA targeting the *gal* promoter and 5’-NGG (TGG) as a PAM sequence. In the absence of L-arabinose, both HK1060/pBJ005 and HK1160/pBJ005 showed red colonies on MacConkey D-galactose agar, indicating the uptake and utilization of D-galactose. In the presence of L-arabinose, white colonies were obtained on MacConkey D-galactose agar, which means the blocking of expression of D-galactose metabolizing enzymes by CRISPR interference (CRISPRi).

### Comparison of dCas9 and dCas9-NG on PAM Dependence

**Fig. 2:**
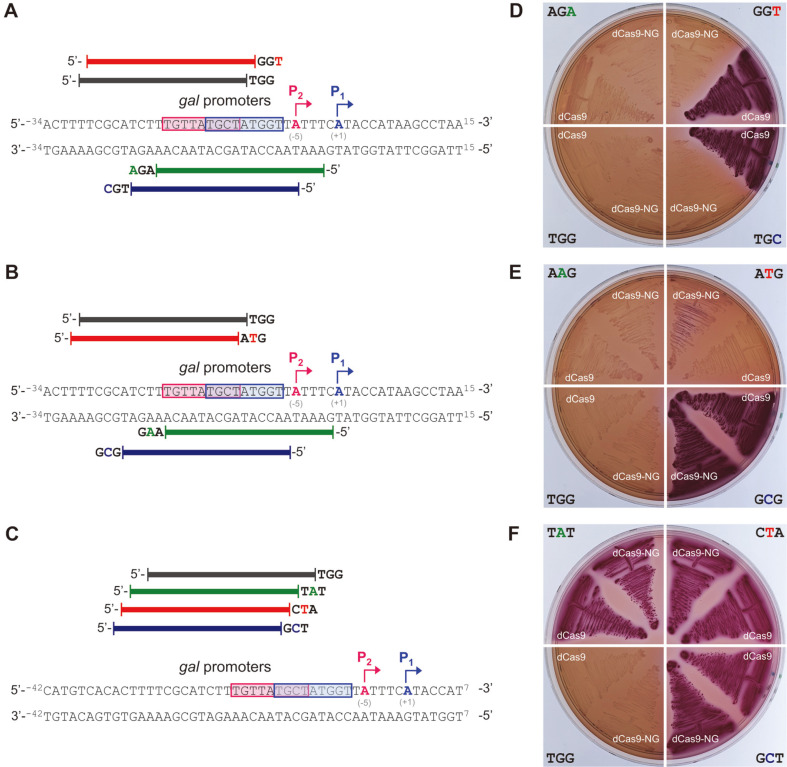
Comparison of PAM dependence between dCas9 and dCas9-NG. Design of target DNA sequences in the *gal* promoter with various PAM sequences: (**A**) 5’-NGN (TGG, AGA, TGC, and GGT); (**B**) 5’-NNG (AAG, GCG, and ATG); (**C**) 5’-NNN (TAT, GCT, and CTA). The dCas9-NG-sgRNA complex was designed to cover the -10 region of the overlapped *gal* *P_1_* and *P_2_* promoters. The blue and red boxes represent the -10 region of each *P_1_* and *P_2_* promoters, respectively. Colony color assay of HK1060 and HK1160 cells carrying the same sgRNA plasmids on MacConkey agar containing both L-arabinose and D-galactose to identify suitable PAM sequences: (**D**) 5’-NGN; (**E**) 5’-NNG; (**F**) 5’-NNN.

Ten sgRNA plasmids targeting the *gal* promoter with various PAM sequences were transformed into HK1060 and HK1160 cells, respectively. Target DNA sequences with various PAMs (5’-NGG, NGN, NNG, and NNN) were designed to inhibit transcription initiation by covering the -10 region of the *gal* promoter (Figs. 2A-2C). We compared dCas9 (HK1060) and dCas9-NG (HK1160) on PAM dependence using the same sgRNA plasmids. As a result, white colonies on MacConkey agar containing D-galactose (final 0.5%) and L-arabinose (1 mM) means that dCas9-NG can repress DNA targets with any 5’-NGN (TGG, AGA, TGC, and GGT) as a PAM sequence (Fig. 2D). However, red colonies showed that dCas9 cannot repress the DNA target with 5’-NGC (TGC), and NGT (GGT) as a PAM sequence. Colony color assay was expanded to 5’-NNG (AAG, GCG, and ATG) (Fig. 2E). As a result, in case of 5’-AAG, both dCas9 and dCas9-NG can repress D-galactose metabolism (white colonies). However, in case of 5’-GCG, both dCas9 and dCas9-NG cannot inhibit D-galactose metabolism (red colonies). In case of 5’-ATG, slight utilization of D-galactose was observed in dCas9-NG (pink colonies). In addition, when three random 5’-NNN was tested, red colonies showed that none of 5’-TAT, GCT, and CTA can repress the *gal* promoter (Fig. 2F).

### Control of D-Galactose Metabolic Rates by dCas9-NG-sgRNA Complexes

**Fig. 3:**
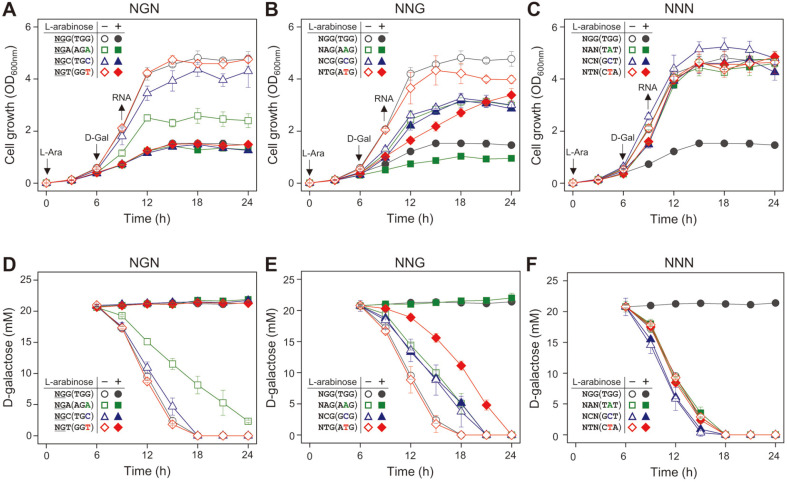
Effect of various PAM sequences recognized by dCas9-NG on cell growth and D-galactose consumption. Cell growth rates (**A**, **B**, and **C**), and D-galactose consumption rates (**D**, **E**, and **F**) were monitored in the presence or absence of L-arabinose that is required for the formation of dCas9-NG-sgRNA complex. L-arabinose was added at the beginning of the culture. D-galactose was added at 6 h, and sampling for RNA extraction was performed at 9 h. (**A**) and (**D**) 5’-NGN; (**B**) and (**E**), 5’-NNG; (**C**) and (**F**), 5’-NNN.

HK1160 cells carrying each sgRNA plasmids were grown in M9 minimal medium containing succinate (final 20 mM) with or without L-arabinose (1 mM). Six hours after the beginning of the cultures (~OD_600_ of 0.3), D-galactose (final 20 mM) was added in the culture. As a result, in case of 5’-NGN (TGG, AGA, TGC, and GGT) as a PAM sequence, D-galactose cannot be consumed at all in the presence of L-arabinose (Figs. 3A and 3D). These results indicate that those dCas9-NG-sgRNA complexes can tightly bind to the *gal* promoter, and inhibit the expression of D-galactose metabolizing enzymes. In case of 5’-AGA, cell growth and D-galactose consumption was slightly retarded even in the absence of L-arabinose, which might be due to the presence of sgRNA transcribed by the constitutive *P_J23119_* promoter in the cell. Among 5’-NNG (AAG, GCG, and ATG) as a PAM sequence, only 5’-AAG was fully functional as a PAM. In case of 5’-ATG, D-galactose was slowly consumed even in the absence of L-arabinose (Figs. 3B and E), which is consistent with the result of pink colonies in MacConkey agar containing both D-galactose and L-arabinose. Lastly, in case of 5’-NNN (TAT, GCT, and CTA), we could not observe any difference in cell growth and D-galactose consumption in presence or absence of L-arabinose. This means that dCas9-NG-sgRNA complex recognizing 5’-NNN as a PAM sequence is not functional at all.

**Fig. 4:**
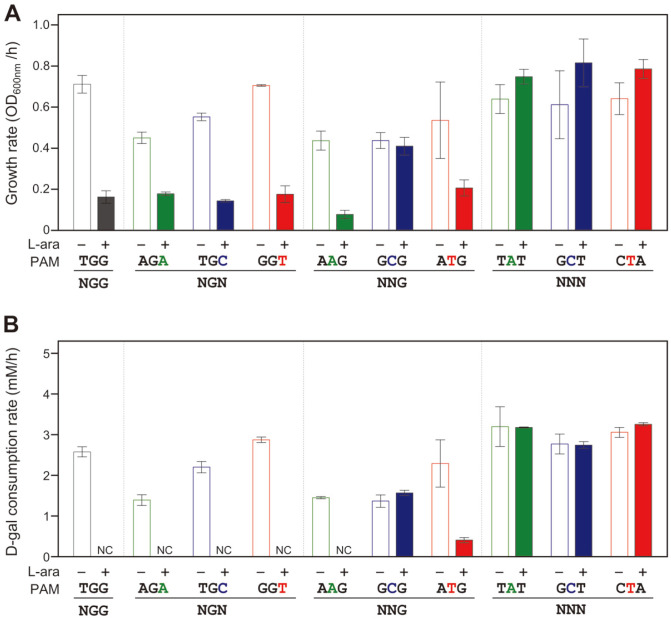
Comparison of (A) cell growth rates (OD_600nm_/h) and (B) D-galactose consumption rates (mM/h) according to various PAM sequences in CRISPRi. Changes of OD_600nm_ and residual D-galactose concentrations between 9 and 12 h were used for the calculation of growth rates and D-galactose consumption rates, respectively. NC, not consumed.

When the formation of dCas9-NG-sgRNA complex was achieved by L-arabinose, the growth rates of 5’-NGN (TGG, AGA, TGC, and GGT) plus 5’-NAG (AAG) decreased below 0.18 (Fig. 4A), and any D-galactose cannot be consumed (Fig. 4B). In case of 5’-ATG as a PAM, the repression of the *gal* promoter is incomplete as mentioned above. In case of 5’-NCG (GCG), and NNN (TAT, GCT, and CTA), growth rates and D-galactose consumption were not affected by CRISPRi.

### The Repression of *gal* Transcription by dCas9-NG Mediated CRISPRi

**Fig. 5:**
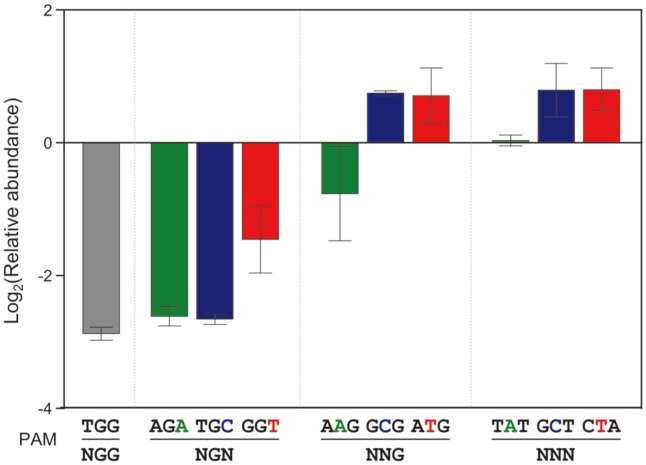
Relative transcript analysis of *galE* gene affected by the presence of dCas9-NG-sgRNA complexes. The relative abundance of *galE* transcripts was obtained by calculating the normalized transcript level of *galE* gene in the presence of L-arabinose (*dcas9-NG* induced) divided by those in the absence of L-arabinose (*dcas9-NG* uninduced).

The dCas9-NG-sgRNA complex can recognize proper PAM sequences, and bind to the *gal* promoter to inhibit transcription initiation. RT-qPCR analysis was used to measure the level of *gal* transcription by targeting the *galE* gene, the first structural gene in the *gal* operon. In case of 5’-NGG (TGG), and NGN (AGA, and TGC) as a PAM sequence, the transcription of *galE* was significantly repressed by the dCas9-NG-sgRNA complex. In case of 5’-NGT (GGT) and NAG (AAG), the transcription was also affected by dCas9-NG mediated CRISPRi. In other PAM sequences, no repression of the *gal* transcription was observed in the presence of L-arabinose (Fig. 5).

## Discussion

The dCas9 protein attaches to the target sequence such as promoter and inhibits gene expression by interfering with RNA polymerase transcription, which is called CRISPRi [18]. CRISPRi-mediated gene regulation can be advantageously used, because the switch can be turned on/off at the desired point in time [25]. However, PAM sequences can be an obstacle when selecting target DNA in various CRISPR technologies including CRISPRi. Cas9-NG has been reported to be able to operate not only 5’-NGG, but also NGN (NGA, NGT, NGC) as PAM [16]. In vitro binding assay showed that operate as 5’-NGG and NGA operate as PAM in common in dCas9 and dCas9-NG, and 5’-NGT operates as a PAM only in dCas9-NG [26]. However, possible combinations of PAM sequences such as NNG (NAG, NCG, NTG) have not been studied in the previous reports.

In our study, possible PAM sequences (5’-NGG, NGN, NNG, and NNN) were systematically compared in dCas9 and dCas9-NG. We designed controllable CRISPRi system in *E. coli*, which consists of L-arabinose-inducible *dcas9-NG* gene in the chromosome and sgRNA that is expressed under control of the constitutive *P_J23119_* promoter in the plasmid (Fig. 1A). In order to test which PAM sequence is suitable for dCas9-NG, sgRNA targeting the *gal* promoter was constructed (Fig. 1B). On MacConkey agar containing D-galactose and L-arabinose, we found that *E. coli* colonies are white because cells cannot utilize D-galactose due to the L-arabinose-induced dCas9-NG-sgRNA complex targeting the *gal* promoter (Fig. 1C).

Ten cases of PAM sequences (5’-NGN, NNG, and NNN) were compared in dCas9 and dCas9-NG using MacConkey D-galactose agar (Fig. 2). In Cas9, it has been reported that NGG as well as NGA and NAG can also operate as PAM [16, 27]. In addition to 5’-NGA, and NAG, our previous work showed that NTG can be played as a PAM sequence in dCas9 [23]. The result of colony color assay was consistent with the previous reports that 5’-NGG, NGA, NAG, and NTG can operate as a PAM sequence in dCas9 (Figs. 2D and E).

While 5’-NGC (TGC) and NGT (GGT) did not work as a PAM in dCas9, it was confirmed to work as a PAM in dCas9-NG (Fig. 2D). In case of 5’-NGA (AGA), cell growth was retarded even in the absence of L-arabinose (Fig. 3A), indicating the possibility that sgRNA itself interferes with the transcription derived from the *gal* promoter. However, transcript analysis showed that the transcript level of *galE* gene in the case of 5’-NGA (AGA) was similar to those in 5’-NGG (TGG) and NGC (TGC) (Fig. 5). The reason is not clear, but the presence of sgRNA can somehow interfere with the D-galactose metabolism.

In the case of 5'-NAG (AAG), a decrease in cell growth rate was observed in the presence of L-arabinose (Fig. 3B). This shows that the dCas9-NG-sgRNA complex can not only inhibit D-galactose metabolism, but also affect whole cell metabolism. It is assumed that dCas9-NG-sgRNA complex can operate where it is not the target. Colony color assay showed that 5’-NTG (ATG) worked as a suitable PAM in dCas9, but incompletely in dCas9-NG (Fig. 2E). RT-qPCR experiments showed 5’-NTG (ATG) cannot be properly recognized as a PAM sequence in dCas9-NG (Fig. 5).

In summary, our study systematically explored the tolerance of PAM when the dCas9-NG-sgRNA complex binds to the target DNA. Colony color assay showed that four nucleotide sequences (5'-NGG, NGA, NAG, and NTG) can act as PAM sequences in dCas9. We confirmed that five nucleotide sequences (5'-NGG, NGA, NGC, NGT, and NAG) can work as PAM sequences in dCas9-NG, by comparing gene expression levels and analyzing D-galactose metabolism. When dCas9-NG, which has an expanded PAM sequence compared to dCas9, is used for CRISPRi, the restrictions on target selection can be alleviated and gene expression can be tightly controlled.

## Supplemental Material



Supplementary data for this paper are available on-line only at http://jmb.or.kr.

## Figures and Tables

**Table 1:** Strains and plasmids used in this study.

Name	Characteristics	Source/reference
Strain		
DH5α	*fhuA2 lac(del)U169 phoA glnV44 Φ80' lacZ(del)M15 gyrA96 recA1 relA1 endA1 thi-1 hsdR17*	Laboratory stock
MG1655	F^−^ *ilvG rfb*-50 *rph*-1	S. Adhya
HK1060	MG1655, Δ*araBAD*::P_BAD_-*dcas9*-KmR	[23]
HK1160	MG1655, Δ*araBAD*::P_BAD_-*dcas9-NG*-KmR	This study
Plasmid		
pKD46	pSC101*ori*^ts^, *araC*, λ *red* genes, AmpR	[28]
pBJ005	PAM(TGG), pBR322 *ori*, sgRNA target ^-30^TTCGCATCTTTGTTATGCTA^-11^ in P_*gal*_), SpR	[23]
pBJ027	PAM(AAG), pBR322 *ori*, sgRNA target ^-1^GAAATAACCATAGCATAACA^-20^ in P_*gal*_), SpR	[23]
pBJ028	PAM(GCG), pBR322 *ori*, sgRNA target ^-6^AACCATAGCATAACAAAGAT^-25^ in P_*gal*_ ), SpR	[23]
pBJ029	PAM(ATG), pBR322 *ori*, sgRNA target ^-31^TTTCGCATCTTTGTTATGCT^-12^ in P_*gal*_ ), SpR	[23]
pBJ030	PAM(AGA), pBR322 *ori*, sgRNA target ^-2^AAATAACCATAGCATAACAA^-21^ in P_*gal*_), SpR	[23]
pBJ031	PAM(TGC), pBR322 *ori*, sgRNA target ^-5^TAACCATAGCATAACAAAGA^-24^ in P_*gal*_ ), SpR	[23]
pBJ032	PAM(GGT), pBR322 *ori*, sgRNA target ^-29^TCGCATCTTTGTTATGCTAT^-10^ in P_*gal*_ ), SpR	[23]
pBJ035	PAM(GCT), pBR322 *ori*, sgRNA target ^-34^ACTTTTCGCATCTTTGTTAT^-15^ in P_*gal*_ ), SpR	This study
pBJ036	PAM(CTA), pBR322 *ori*, sgRNA target ^-33^CTTTTCGCATCTTTGTTATG^-14^ in P_*gal*_ ), SpR	This study
pBJ037	PAM(TAT), pBR322 *ori*, sgRNA target ^-32^TTTTCGCATCTTTGTTATGC^-13^ in P_*gal*_ ), SpR	This study

**Table 2:** Primers and synthetic DNA used in this study.

Name	Sequence (5’→3’)	Description
P1	GCTTTTTATCGCAACTCTCTACTGTTTCTCCATACCCGTTTTTTTGGATGGAGTGAAACGATGGATAAGAAATACTCAATAGGCT	Construction of *dcas9-NG*-KmR cassette
P2	GACAATATTGACTTGGGGCATGGACAATACTTTG	
P3	GCTAGGAGGTGACTGAATTCCGGGGATCCGTCGACCTGCAG	
P4	GTGGTGCCGGTTGCTGGAATCGACTGACCCGCCTGCGCCCAGATGGTGGCGTGGCGCGAGTG TAGGCTGGAGCTGCTTCGAAGTT	
P5	GATACTGGGCCGGCAGGCGCTCCATTGCCC	Common primer for sgRNA plasmid
P6	ACTTTTCGCATCTTTGTTATGTTTTAGAGCTAGAAATAGCAAG	pBJ035 plasmid
P7	GCAATGGAGCGCCTGCCGGCCCAGTATCAG	Common primer for sgRNA plasmid
P8	ATAACAAAGATGCGAAAAGTACTAGTATTATACCTAGGACTG	pBJ035 plasmid
P9	CTTTTCGCATCTTTGTTATGGTTTTAGAGCTAGAAATAGCAAG	pBJ036 plasmid
P10	CATAACAAAGATGCGAAAAGACTAGTATTATACCTAGGACTG	pBJ036 plasmid
P11	TTTTCGCATCTTTGTTATGCGTTTTAGAGCTAGAAATAGCAAG	pBJ037 plasmid
P12	GCATAACAAAGATGCGAAAAACTAGTATTATACCTAGGACTG	pBJ037 plasmid
P13	CCACCGTTTATGGCGATCAG	qPCR for *galE*
P14	GTTCCACCATCAGCTTGCTT	
P15	CAGCAGCCGCGGTAATAC	qPCR for 16S rRNA
P16	ACCAGGGTATCTAATCCTGT	
