# Supplementary material for: Effective Blocking of Microbial Transcriptional Initiation by dCas9-NG-Mediated CRISPR Interference
Source: J Microbiol Biotechnol. 2020 Sep 22;30(12):1919–26. doi: 10.4014/jmb.2008.08058 (PMC9728369; doi:10.4014/jmb.2008.08058)
Supplement: Supplementary file 1 [file JMB-30-12-1919-supple.pdf]

https://doi.org/10.4014/jmb.2008.08058

Supplementary Information

Nucleotide sequence of codon optimized partial DNA fragment of dCas9-NG (837 bp)

CAAGTCAATATTGTCAAGAAAACAGAAGTACAGACAGGCGGATTCTCCAAGGAGTC

AATTCGCCCAAAAAGAAATTCGGACAAGCTTATTGCTCGTAAAAAAGACTGGGATCC

AAAAAAATATGGTGGTTTTGTGAGTCCAACGGTAGCTTATTCAGTCCTAGTGGTTGCT

AAGGTGGAAAAAGGGAAATCGAAGAAGTTAAAATCCGTTAAAGAGTTACTAGGGAT

CACAATTATGGAAAGAAGTTCCTTTGAAAAAAATCCGATTGACTTTTTAGAAGCTAA

AGGATATAAGGAAGTTAAAAAAGACTTAATCATTAAACTACCTAAATATAGTCTTTTT

GAGTTAGAAAACGGTCGTAAACGGATGCTGGCTAGTGCCCGCTTCTTACAAAAAGG

AAATGAGCTGGCTCTGCCAAGCAAATATGTGAATTTTTTATATTTAGCTAGTCATTATG

AAAAGTTGAAGGGTAGTCCAGAAGATAACGAACAAAAACAATTGTTTGTGGAGCAG

CATAAGCATTATTTAGATGAGATTATTGAGCAAATCAGTGAATTTTCTAAGCGTGTTAT

TTTAGCAGATGCCAATTTAGATAAAGTTCTTAGTGCATATAACAAACATAGAGACAAA

CCAATACGTGAACAAGCAGAAAATATTATTCATTTATTTACGTTGACGAATCTTGGAG

CTCCCCGCGCTTTTAAATATTTTGATACAACAATTGATCGTAAAGTGTATCGCTCTACA

AAAGAAGTTTTAGATGCCACTCTTATCCATCAATCCATCACTGGTCTTTATGAAACAC

GCATTGATTTGAGTCAGCTAGGAGGTGACTGA
